# Metabolomics Analysis Identifies Intestinal Microbiota-Derived Biomarkers of Colonization Resistance in Clindamycin-Treated Mice

**DOI:** 10.1371/journal.pone.0101267

**Published:** 2014-07-02

**Authors:** Robin L. P. Jump, Alex Polinkovsky, Kelly Hurless, Brett Sitzlar, Kevin Eckart, Myreen Tomas, Abhishek Deshpande, Michelle M. Nerandzic, Curtis J. Donskey

**Affiliations:** 1 Geriatric Research Education and Clinical Center, Cleveland Veterans Affairs Medical Center, Cleveland, Ohio, United States of America; 2 Division of Infectious Diseases and HIV Medicine, Department of Medicine, Case Western Reserve University, Cleveland, Ohio, United States of America; 3 Research Service, Cleveland Veterans Affairs Medical Center, Cleveland, Ohio, United States of America; Universidad Andres Bello, Chile

## Abstract

**Background:**

The intestinal microbiota protect the host against enteric pathogens through a defense mechanism termed colonization resistance. Antibiotics excreted into the intestinal tract may disrupt colonization resistance and alter normal metabolic functions of the microbiota. We used a mouse model to test the hypothesis that alterations in levels of bacterial metabolites in fecal specimens could provide useful biomarkers indicating disrupted or intact colonization resistance after antibiotic treatment.

**Methods:**

To assess *in vivo* colonization resistance, mice were challenged with oral vancomycin-resistant *Enterococcus* or *Clostridium difficile* spores at varying time points after treatment with the lincosamide antibiotic clindamycin. For concurrent groups of antibiotic-treated mice, stool samples were analyzed using quantitative real-time polymerase chain reaction to assess changes in the microbiota and using non-targeted metabolic profiling. To assess whether the findings were applicable to another antibiotic class that suppresses intestinal anaerobes, similar experiments were conducted with piperacillin/tazobactam.

**Results:**

Colonization resistance began to recover within 5 days and was intact by 12 days after clindamycin treatment, coinciding with the recovery bacteria from the families *Lachnospiraceae* and *Ruminococcaceae*, both part of the phylum *Firmicutes*. Clindamycin treatment caused marked changes in metabolites present in fecal specimens. Of 484 compounds analyzed, 146 (30%) exhibited a significant increase or decrease in concentration during clindamycin treatment followed by recovery to baseline that coincided with restoration of *in vivo* colonization resistance. Identified as potential biomarkers of colonization resistance, these compounds included intermediates in carbohydrate or protein metabolism that increased (pentitols, gamma-glutamyl amino acids and inositol metabolites) or decreased (pentoses, dipeptides) with clindamycin treatment. Piperacillin/tazobactam treatment caused similar alterations in the intestinal microbiota and fecal metabolites.

**Conclusions:**

Recovery of colonization resistance after antibiotic treatment coincided with restoration of several fecal bacterial metabolites. These metabolites could provide useful biomarkers indicating intact or disrupted colonization resistance during and after antibiotic treatment.

## Introduction

The gastrointestinal tract of adult mammals is inhabited by a complex microbial community that includes hundreds of distinct bacterial species [1]–[3]. The intestinal microbiota can be classified into 4 principal phyla: *Firmicutes* and *Actinobacteria* (Gram-positive) and *Bacteroidetes* and *Proteobacteria* (Gram-negative), with *Firmucutes* and *Bacteroidetes* typically being dominant in healthy mammals [4]. The microbiota complement host physiology by providing a rich array of metabolic functions that benefit the host [5]. Key functions provided by intestinal microorganisms include bile salt metabolism, synthesis of vitamins, digestion and fermentation of otherwise non-digestible polysaccharides and proteins, and stimulation of immune function [1], [5].

The indigenous microbiota of the colon also provide a critical host defense by inhibiting growth of potentially pathogenic microorganisms. This defense mechanism, termed colonization resistance, can be applied to prevention of colonization by exogenously introduced organisms and to prevention of overgrowth of resident bacteria usually present in low numbers [5], [6]. Antibiotics excreted into the intestinal tract may disrupt colonization resistance, leaving the host vulnerable to infection with pathogens such as *Clostridium difficile* and vancomycin-resistant enterococci (VRE) [7], [8]. Although the organisms that establish and maintain colonization resistance are not known, several recent studies have identified specific bacterial species or combinations of species that may be involved. In antibiotic-treated mice, partial restoration of colonization resistance to *Clostridium difficile* and VRE was attained through administration of isolates from the bacterial families *Lachnospiraceae* (phylum *Firmucutes*, clostridial cluster XIVa) [9] and *Barnsiella* (phylum *Bacteroidetes*) [10], respectively. Lawley *et al*. demonstrated that a mixture of six phylogenetically diverse intestinal bacteria restored colonization resistance to *C. difficile* in mice, suggesting that synergistic action of multiple organisms might be required [11].

Although many studies have examined antibiotic-induced changes in the intestinal microbiota, limited information is available on the effect of antibiotic treatment on bacterial metabolites. Some studies have suggested that levels of short chain fatty acids produced by bacterial metabolism could be an indicator or mediator of colonization resistance [11], [12]. However, it is not known if specific profiles of bacterial metabolites are associated with intact colonization resistance. Here, we used a mouse model to test the hypothesis alterations in levels of bacterial metabolites in fecal specimens could provide useful biomarkers indicating disrupted or intact colonization resistance after antibiotic treatment. After treatment with the lincosamide antibiotic clindamycin, the timing of recovery of *in vivo* colonization resistance was determined by challenging mice with *C. difficile* spores or VRE. Non-targeted metabolic profiling by gas chromatograph (GC)/mass spectrometry (MS) and ultra-high performance liquid chromatography-tandem MS (UPLC-MS/MS) to identify fecal metabolites associated with disrupted versus intact colonization resistance and quantitative real-time PCR (qPCR) was performed to evaluate concurrent changes in the microbiota. To assess whether the findings were applicable to another antibiotic class that suppresses intestinal anaerobes, similar experiments were conducted with the beta-lactam/beta-lactamase inhibitor antibiotic piperacillin/tazobactam.

## Materials and Methods

### Ethics Statement

The Animal Care Committee of the Cleveland Veterans Affairs Medical Center approved the study protocol. The VRE test strain was isolated from a patient at the Cleveland VA Medical Center. The Institutional Review Board of the Cleveland VA Medical Center approved the study protocol for collection of the isolate. Informed consent was not obtained because the isolate was cultured from clinical samples with no collection of patient identifiers or interaction with the subject.

### Bacterial Strains

*E. faecium* C68 is a clinical VanB VRE isolate that has been used in previous mouse colonization studies [7]. For C68, the minimum inhibitory concentrations (MICs) of clindamycin and piperacillin/tazobactam are >10,000 µg/mL and 1,250 µg/mL, respectively [7]. VA17 is an epidemic North American pulsed-field gel electrophoresis type 1 (NAP1) *C. difficile* strain. For VA17, the MICs of clindamycin and piperacillin/tazobactam are 128 µg/mL and 2 µg/mL, respectively [8]. *C. difficile* spores were prepared as previously described [13].

### *In vivo* Mouse Model of Colonization Resistance

We used a mouse model we described previously to evaluate recovery of colonization resistance after antibacterial treatment [7]; in this model, mice do not develop weight loss or other overt evidence of illness due to antibiotic treatment or colonization by pathogens, including toxigenic *C. difficile*. Female CF-1 mice weighing 25 to 30 g (Harlan Sprague-Dawley, Indianapolis, IN) were housed in individual micro-isolator cages. Mice received daily subcutaneous injections (0.2-mL total volume) of saline, clindamycin (1.4 mg/day) for 3 days. The antibiotic dose was equal to the usual human doses administered over a 24-hour period (milligrams of antibiotic per gram of body weight). To assess *in vivo* colonization resistance, mice were challenged with 10^4^ colony-forming units (CFU) of VRE C68 or *C. difficile* VA17 spores by orogastric gavage before and 1, 5, and 12 days after completion of antibiotic treatment (3 saline controls and 6 clindamycin-treated mice per group were included for each time point). Fresh stool specimens were collected 2 and 4 days after gavage and the concentration of pathogens was measured by plating serially diluted samples on selective agar as previously described [7]. Pre-reduced cycloserine-cefoxitin-brucella agar containing 0.1% taurocholic acid and lysozyme 5 mg/mL (CDBA) [13] and Enterococcosel agar (Becton Dickinson, Sparks, MD) containing 20 µg/mL of vancomycin were used as selective media for *C. difficile* and VRE, respectively. Colonization resistance was deemed intact at a given time point if there was no significant increase in concentrations of the pathogens in the stool of antibiotic-treated mice at 4 days post-challenge in comparison to the control mice. The experiments were performed in duplicate.

### Measurement of Antibiotic Concentrations in Feces

Mice were given clindamycin as described above. The concentration of the antibiotics in stool specimens collected on day 3 of antibiotic treatment was measured using an agar well diffusion assay with *Clostridium perfringens* as the indicator strain [14].

### Microbiota Analysis

Fecal specimens were collected from mice given clindamycin (n = 6) or saline (n = 3), as described above, at 1, 5, 8, 12 and 21 days after the final antibiotic dose for analysis of the intestinal microbiota and stool metabolites. qPCR was performed using the methods of Louie *et al*. [15]. The primers used and their bacterial targets are shown in Table 1 [15]–[22]. Fecal specimens were frozen at −80°C prior to analysis. Fecal bacterial DNA was extracted from 100 mg of feces using the QIAmp DNA Stool Mini Kit (Qiagen, Hilden, Germany) according to the manufacturer's instructions. DNA was eluted from the columns with 200 µL of nuclease-free ultrapure molecular biology grade water. The concentration of bacterial DNA in nanograms per μL was measured using the Qubit 2.0 Fluorometer (Life Technologies, Carlsbad, CA).

**Table 1:** 16S Ribosomal RNA probes used for quantitative real-time polymerase chain reaction to quantify changes in components of the fecal microbiota during and after treatment with clindamycin.

Bacterial Target Family^a^	Bacterial Target Genus & Species^a^	Primer Name	Sequence (5′ → 3′)	Standard	Reference
*Bacteroidaceae, Prevotellaceae*	*Bacteroides* spp.	Uni331F	TCCTACGGGAGGCAGCAGT	*Bacteroides fragilis*	[17]
		Bac708R	CAATCGGAGTTCTTCGTG		[18]
*Enterobacteriaceae*	*Enterobacter* spp.	Eco1457F	CATTGACGTTACCCGCAGAAGAAGC	*Escherichia coli*	[19]
		Eco1652R	CTCTACGAGACTCAAGCTTGC		[19]
*Ruminococcaceae*	*Ruminococcus, Faecalibacterium, Ethanoligenes* spp. and *Clostridiales leptum* subgroup	sg-Clept-F	GCACAAGCAGTGGAGT	*Clotridium leptum*	[20]
		sg-Clept-R	CTTCCTCCGTTTTGTCAA		[20]
*Lachnospiraceae*	*Blautia, Pseudobutyrivibrio, Roseburia* spp. and *Clostridiales coccoides* group	Erec482R	GCTTCTTAGTCARGTACCG	*Ruminoccus torques*	probeBasepB-00963 [22]
		Eub338F	ACTCCTACGGGAGGCAGC		probeBasepB-00159 [22]
*Desulfovibrio-naeceae*	*Desulfovibro* spp.	g-desulf-F	GGTACCTTCAAAGGAAGCAC	*Desulfovibrio desulfuricans*	[16]
		g-desulf-R	GGGATTTCACCCCTGACTTA		[16]
*Enterococcaceae*	*Enterococcus* spp.	g-enter-F	CCCTTATTGTTAGTTGCCATCATT	*Enterococcus faecium*	[16]
		g-enter-R	ACTCGTTGTACTTCCCATTGT		[16]
*Veillonellaceae*	*Veillonella* spp.	g-veill-F	A(C/T)CAACCTGCCCTTCAGA	*Veillonella parvula*	[16]
		g-veill-R	CGTCCCGATTAACAGAGCTT		[16]
*Prevotellaceae*	*Prevotella* spp.	CFB286F	GTAGGGGTTCTGAGAGGA	*Prevotella oris*	probeBase pB-00045 [22]
		CFB719R	AGCTGCCTTCGCAATCGG		probeBase pB-00047 [22]
*Lactobacillaceae, Planococcaceae*	*Lactobacillus* spp.	Uni331F	TCCTACGGGAGGCAGCAGT	*Lactobacillus acidophilus*	[17]
		Lacto371R	TGGAAGATTCCCTACTGC		probeBase pB-00195 [22]
*Bifidobacteriaceae*	*Bifidobacterium* spp.	Bif551F	CGCGTCYGGTGTGAAAG	*Bifidobacterium* spp	[21]
		Bif794R	CCCCACATCCAGCATCCA		[21]
*Eubacteria*	Total bacteria	515F	GTGCCAGCAGCCGCGGTAA	*B. fragilis* &*E. coli*	[50]
		685R	TCTACGCATTTCACCGCTAC		

a as determined using TestProbe from SILVA-ARB (www.silva-arb.de) [23].

Bacterial targets for each primer set were determined *in silico* using using TestProbe from SILVA-ARB (www.silva-arb.de) [23]. The results for each primer set were concatenated using R to identify amplicons from known bacterial species [24]. Sequences representing 1% or more of the amplicons are in shown in Table S1 and form the basis for the Bacterial Target columns in Table 1. The specificity of the primers for DNA obtained from bacteria used for standards was assessed using the methods of Louie *et al.* [15]. Reference strains are shown in Table 1. Purified template DNA from the reference strains was used for melting curve analysis and to generate standard curves for each primer set using 10-fold serial dilutions of DNA ranging from 10 to 10^−6^ ng. The optimum annealing temperature and specificity of each primer set was determined using a Mastercycler pro (Eppendorf, Hamburg, Germany) and 2X PCR Master Mix (Promega, Fitchburg, WI) with gradient. qPCR was performed using the CFX96 detection system (Biorad, Hercules, CA). Amplification and detection were conducted in 96-well plates with SYBR Green 2× qPCR Master Mix (BioRad). Each sample was run in triplicate in a final volume of 20 µl containing a final concentration of 0.3 µM of each primer and 5 µL of template DNA using the following parameters: 1 cycle at 94°C for 5 minutes, followed by 49 cycles at 94°C for 20 seconds, 56°C–58°C for 20 seconds, and 72°C for 20 seconds.

To evaluate the correlation between qPCR and culture, fresh fecal specimens collected at the same time points were used for quantitative culture of enterococci. The specimens were emulsified in 5-fold (weight/volume) pre-reduced phosphate buffered saline. Serially diluted aliquots were inoculated onto Enterococcosel agar. Plates were incubated at 37°C for 48 hours and CFU per gram of stool were calculated.

### Fecal Metabolite Analysis

Analysis of metabolic compounds in fecal specimens was conducted by Metabolon (Durham, NC) using methods described previously [25]–[27]. Fecal samples underwent a methanol extraction under vigorous shaking for 2 min (Glen Mills Genogrinder 2000) to remove the protein fraction while maximizing recovery of small molecules. The resulting extract was divided into fractions for analysis using GC/MS, UPLC-MS/MS (positive mode), and UPLC-MS/MS (negative mode).

The UPLC-MS/MS platform utilized an Acquity UPLC (Waters, Milford, CA) with Waters UPLC BEH C18-2.1×100 mm, 1.7 µm columns and a ThermoFisher LTQ mass spectrometer, which included an electrospray ionization source and a linear ion-trap mass analyzer. The instrumentation was set to monitor for positive ions in acidic extracts or negative ions in basic extracts through independent injections. The instrument was set to scan 99–1000 m/z and alternated between MS and MS/MS scans. Samples destined for analysis by GC-MS were dried under vacuum desiccation for a minimum of 18 hours prior to being derivatized using bis(trimethylsilyl)trifluoroacetamide. Derivatized samples were separated on a 5% phenyldimethyl silicone column with helium as carrier gas and a temperature ramp from 60° to 340°C within a 17-min period. All samples were analyzed on a Thermo-Finnigan Trace DSQ fast-scanning single-quadrupole MS operated at unit mass resolving power with electron impact ionization and a 50–750 atomic mass unit scan range.

Metabolites were identified by automated comparison of the ion features in the experimental samples to a reference library of chemical standard entries that included retention time, molecular weight (m/z), preferred adducts, and in-source fragments as well as associated MS spectra and curated by visual inspection for quality control using software developed at Metabolon [26]. Identification of known chemical entities was based on comparison to metabolomic library entries of more than 2,400 purified standards. Peaks were quantified using area-under-the-curve. Raw area counts for each metabolite in each sample were normalized to correct for variation resulting from instrument inter-day tuning differences by the median value for each run-day, therefore, setting the medians to 1.0 for each run. This preserved variation between samples but allowed metabolites of widely different raw peak areas to be compared on a similar graphical scale. Missing values were imputed with the observed minimum after normalization.

### Evaluation of Colonization Resistance, Fecal Microbiota, and Fecal Metabolites in Mice Treated with Piperacillin/Tazobactam

To assess the applicability of the findings for clindamycin to another antibiotic class, similar experiments were conducted with the beta-lactam/beta-lactamase inhibitor antibiotic piperacillin/tazobactam. Piperacillin/tazobactam suppresses anaerobic intestinal microbiota in a manner similar to clindamycin with disruption of colonization resistance, but also suppresses indigenous enterococci and facultative gram-negative bacilli [7]–[8]. The *in vivo* colonization resistance experiments were identical to the clindamycin experiments with the following exceptions: 3 mice were used for the experimental and control groups at each time point testing recovery of colonization resistance and 4 mice for testing changes in the fecal microbiota and metabolites. The dose of piperacillin/tazobactam was 8 mg/day daily for 3 days. The fecal microbiota analysis and the fecal metabolite analysis conducted by Metabolon was the same as previously described for the clindamycin-treated mice. The library used by Metabolon for the analysis of pipericilln/tazobactam-treated animals differed slightly from the one used for the clindamycin-treated mice and did not include N-acetyl-isoleucine or gamma-glutamylglutmate.

### Data Analysis

Analysis of variance (ANOVA) with repeated measures was used to compare quantities of exogenous bacteria recovered from stool and quantities of DNA from bacterial families for control versus antibiotic-treated mice. For the metabolite analysis, ANOVA with repeated measures was performed after log transformation to identify compounds that differed significantly between control and antibiotic groups. Welch's two-sample *t*-test was used for comparison of the pretreatment groups.

Compounds that differed between antibiotic-treated and control mice were identified as potential biomarkers of colonization resistance if the timing of recovery of the compound to baseline levels correlated closely with the timing of recovery of *in vivo* colonization resistance (i.e., normalization or substantial return toward baseline within 8 days after the final clindamycin dose). Given the large number of comparisons performed in metabolomic discovery studies, it is anticipated that some compounds will show significant differences by random chance. Accordingly, a q-value was used to estimate the false discovery rate [28]. Furthermore, compounds identified as potential biomarkers of colonization resistance were selected for more detailed analysis in the context of other lines of evidence including the Kyoto Encyclopedia of Genes and Genomes (KEGG) database [29] or the Human Metabolome Database [30]. The fold change in the concentrations of the compounds determined to be of importance in distinguishing antibiotic-treated versus control mice in comparison to the baseline levels were graphed over time. Statistical analyses were performed using R [24].

## Results

### Restoration of *in vivo* Colonization Resistance After Clindamycin Treatment

**Figure 1:**
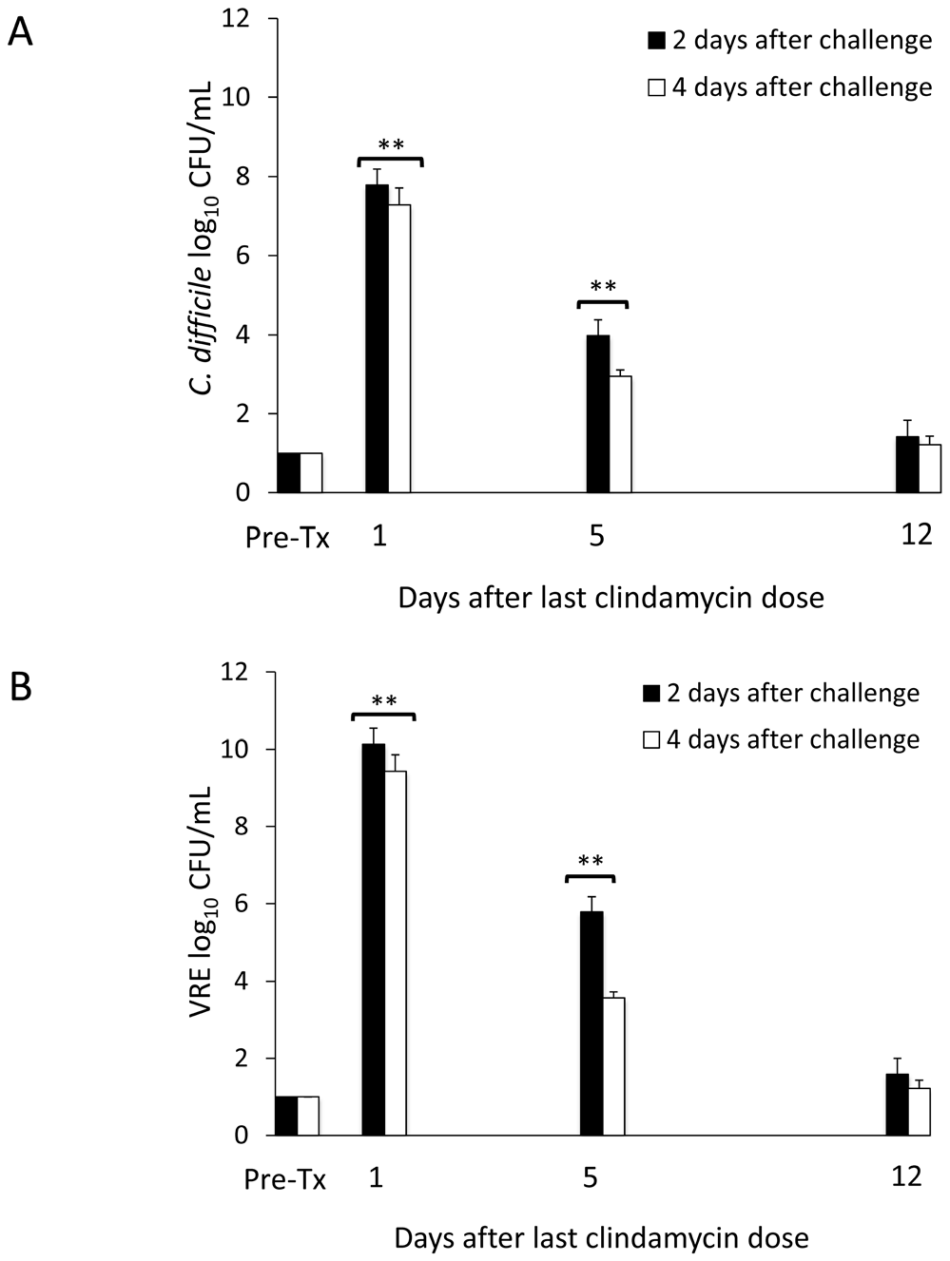
Recovery of *in vivo* colonization resistance over time in clindamycin treated animals. Mice (6 per group at each time point) were challenged with 10^4^ colony-forming units of toxigenic *Clostridium difficile* spores (A) or vancomycin-resistant *Enterococci* (VRE) (B) by orogastric gavage either before treatment or 1, 5, or 12 days following treatment with 3 days of daily subcutaneous clindamycin. Concentrations of the pathogens in feces were measured by quantitative cultures 2 days (black bars) and 4 days (white bars) following pathogen challenge. ** *p*<0.01 compared to other time points. Error bars represent standard error. Results are shown for 1 of 2 duplicate experiments.

**Figure 1** shows the results of the assessment of *in vivo* colonization resistance for 1 of the 2 sets of experiments (similar results were obtained for each set of experiments). In the absence of clindamycin treatment, all baseline and untreated control mice demonstrated intact colonization resistance with either undetectable or only low levels of the pathogens in stool. Clindamycin-treated mice challenged with the pathogens 1 day after the final antibiotic dose developed high-density colonization that remained elevated 4 days later. By 5 days after the final clindamycin dose, mice demonstrated partially restored colonization resistance to both pathogens. By 12 days following clindamycin exposure, the concentrations of *C. difficile* and VRE in stool did not differ from the concentrations in untreated mice, indicating restored colonization resistance. The mean concentration of clindamycin in fecal specimens on day 3 of treatment was 55.9 µg/g (range, 21.2 to 132.2).

### Correlation of Changes in the Intestinal Microbiota with *in vivo* Colonization Resistance

**Figure 2:**
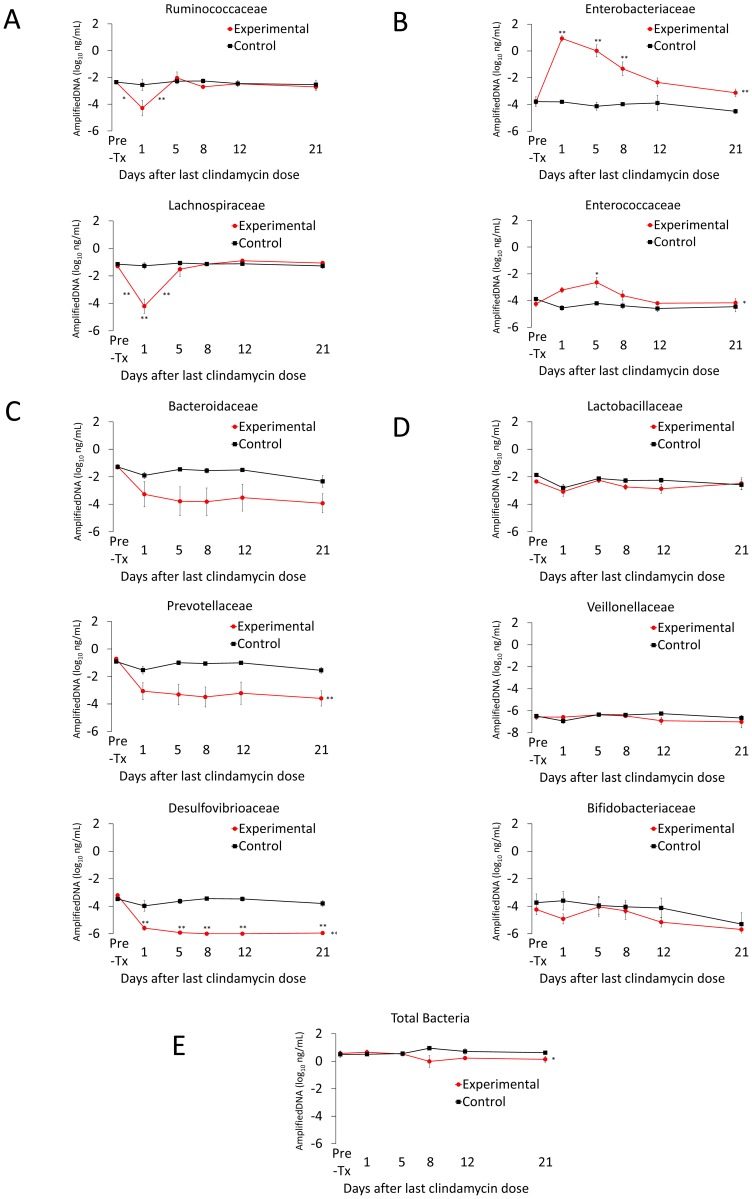
Recovery of the fecal microbiota over time in clindamycin treated animals. Microbiota analysis was performed in conjunction with 1 of the 2 colonization resistance experiments. Mice received subcutaneous clindamycin (n = 6) or normal saline (n = 3) for 3 days. Quantitative real-time PCR was used to measure fecal bacterial DNA in fecal specimens collected either before treatment or 1, 5, 8, 12, or 21 days following treatment. The y-axis shows the quantity of amplified DNA detected per 1 ng of template DNA. The bacterial families with mouse fecal microbiota showed 4 distinct response patterns following clindamycin exposure: (A) decrease followed by a rapid normalization, (B) increase with slow normalization, (C) sustained decrease and (D) no difference compared to controls. Red circles, mean values for clindamycin-treated mice. Black squares, mean values for control mice. * *p*<0.05 and ** *p*<0.001. Symbols adjacent to the line indicate statistically significant differences between consecutive time points. Symbols adjacent to circles indicate statistically significant differences between the experimental and control groups for that individual time point. Symbols after the terminal point of the lines on a graph indicate statistically significant differences between experimental and control groups independent of individual time points. Error bars represent standard error.

**Figure 2** shows the results of qPCR analysis of changes in the microbiota during and after clindamycin treatment. Four distinct response patterns were demonstrated. First, fecal bacterial DNA from the families *Ruminococcaceae* and *Lachnospiraceae* decreased by 1.5 to 3 logs during clindamycin administration with a prompt return to baseline concentrations by 5 days following completion of antibiotics, coinciding with partical recovery of *in vivo* colonization resistance. Second, fecal bacterial DNA from the families *Enterobacteriaceae* and *Enterococcaceae* increased significantly during clindamycin treatment and decreased to or approached the pre-treatment baseline by days 8 to 12 following the final dose of clindamycin, corresponding with recovery of *in vivo* colonization resistance. Third, fecal bacterial DNA from the families *Bacteroidaceae, Prevotellaceae*, and *Desulfovibrioaceae* decreased by ≥ 2 logs during clindamycin administration and failed to recover during the course of the experiment. Finally, concentrations of fecal bacterial DNA from the families *Lactobacilliaceae*, *Veionellaceae* and *Bifidobacteriaceae* were largely unaffected by clindamycin. Quantitative cultures for enterococci correlated well with the qPCR results. The fecal concentration of enterococci increased during clindamycin treatment, decreased by day 8 after the last dose of clindamycin and approached the pre-treatment baseline by day 19 (data not shown).

### Correlation of Fecal Metabolites with *in vivo* Colonization Resistance

Four hundred-eighty four compounds were identified in the fecal samples. **Table S2** shows the metabolites analyzed and the effect of clindamycin treatment expressed as a ratio the metabolites detected in the fecal material of treated animals *vs.* control animals at each time point. Three patterns of response to clindamycin treatment were observed. First, 144 (30%) compounds did not change in concentration in comparison to controls. Second, in clindamycin-treated mice, 24 (5%) compounds exhibited a sustained change compared to controls. Third, 146 (30%) of the compounds demonstrated an initial change during clindamycin treatment followed by a substantial return toward baseline within 8 days after the final clindamycin dose. The remaining compounds showed a variable response to clindamycin treatment (146; 30%) or were different from controls at baseline (24; 5%).

**Figure 3:**
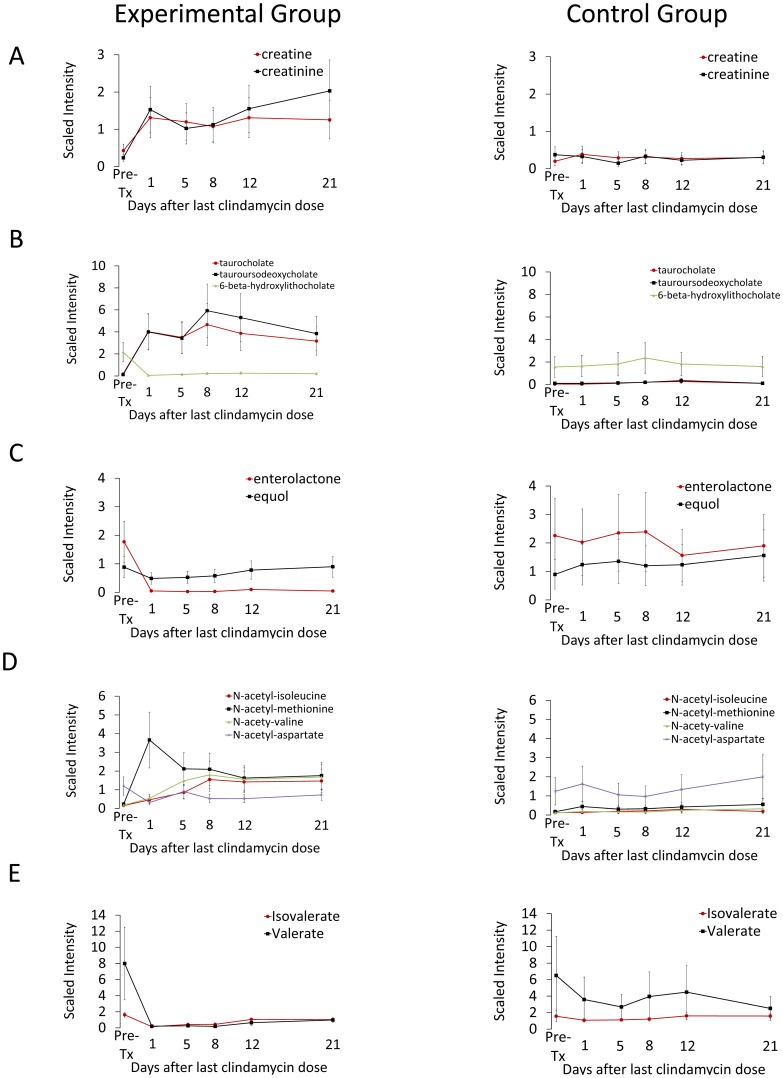
Changes in levels of fecal metabolites of clindamycin-treated mice compared to saline controls for selected compounds that exhibited a sustained increase or decrease after clindamycin treatment. Compounds from pathways related to metabolism of (A) creatinine, (B) bile salts, (C) phytoestrogens, (D) N-acetylated amino acids and (E) short-chain fatty acids. Results from experimental mice are shown on the left and from control animals on the right. Metabolites measured in the experimental group are the significantly different (*p*≤0.05) from the pre-treatment levels at least through day 8 after the final clindamycin dose for all compounds except N-acetyl-aspartate (*p*≤0.10 at days 5, 8; *p*≤0.5 all other times). Error bars represent standard error.

For the second response pattern, 14 (3%) compounds showed a sustained increase and 10 (2%) showed a sustained decrease in experimental *vs.* control animals. Specifically, increases in creatine and creatinine levels following clindamycin treatment persisted throughout the study period (**Figure 3A**), consistent with findings that several members of the gut microbiota metabolize these compounds [31]. Furthermore, clindamycin-treatment corresponded with an increase taurocholate and tauroursodeoxycholate, primary bile acids formed in the liver, and with a decrease in 6-beta-hydroxylithocholate, a secondary bile acid produced through bacterial metabolism of primary bile acids (**Figure 3B**). The levels of these compounds did not return to their respective baseline levels during the study period. Similarly, enterolactone and equol, both products of intestinal bacterial metabolism of phytoestrogens [32], declined following clindamycin treatment; these changes persisted until after recovery of *in vivo* colonization resistance (**Figure 3C**). Furthermore, while three N-acetylated amino acids (methionine, leucine, and isoleucine) increased, N-acetyl-aspartate decreased significantly with clindamycin treatment. None of these amino acids returned to baseline by the end of the study period (**Figure 3D**). Finally, we also examined changes in the short-chain fatty acids (SCFAs) which are produced in the colon through bacterial fermentation of dietary fiber. Fecal levels of valerate and isolvalerate decreased during clindaymycin treatment and failed to normalize during the study period (**Figure 3E**). These compounds were not identified as potential biomarkers or mediators of colonization resistance based upon the absence of correlation with recovery of *in vivo* colonization resistance.

In the third pattern of response, 146 (30%) compounds increased or decreased in concentration during clindamycin treatment followed by normalization or substantial return toward baseline within 8 days after the final clindamycin dose. These compounds, of which 100 (21%) increased and 46 (10%) decreased, are potential biomarkers of colonization resistance based upon the correlation between their recovery and the recovery of *in vitro* colonization resistance. The magnitude of the change or the ability to group compounds into metabolic sub-pathways in which multiple compounds demonstrated similar changes determined which potential biomarkers were selected for further description below.

### 4- and 5-Carbon Sugar Alcohols and Corresponding Sugars

**Figure 4:**
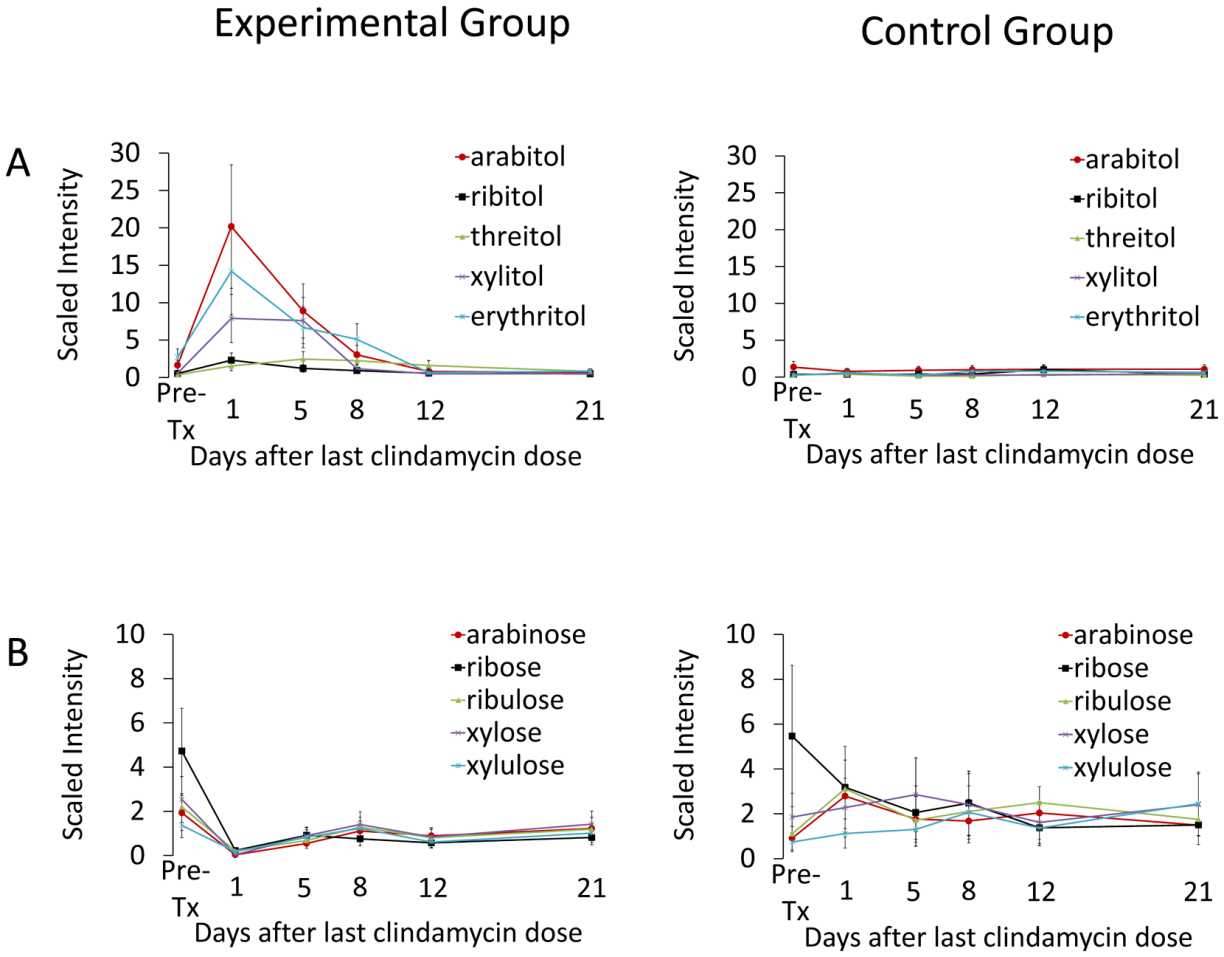
Changes in levels of fecal metabolites of 4- and 5-carbon sugars and alcohols in clindamycin-treated mice compared to saline controls. Compounds from pathways related to metabolism of 4- and 5-carbon (A) alcohols and (B) sugars. Results from experimental mice are shown on the left and from control animals on the right. Metabolites measured in the experimental group are the significantly different (*p*≤0.05) from the pre-treatment levels through day 1 (arabitol, xylulose), day 5 (ribitol, xylitol, arabinose, ribulose, xylose) day 8 (erythirol) or day 12 (threitol, ribose). Error bars represent standard error.

Pentitols (*i.e.*, ribitol, arabitol, xylitol) are 5-carbon sugar alcohols present in many fruits and vegetables. Pentitols and other sugar alcohols are not well absorbed in the small intestine and are metabolized to pentoses by the intestinal microbiota [33]. In addition, mammalian metabolic pathways may convert pentoses to pentitols [34]. The concentration of both 5-carbon (arabitol, ribitol, xylitol) and 4-carbon sugar alcohols (erythritol, threitol) increased significantly during clindamycin treatment, while the concentration of the corresponding pentoses (arabinose, xylose, xylulose, ribose and ribulose) decreased (**Figure 4**). The concentrations of pentitols decreased to baseline by 8-12 days after the final dose of clindamycin, whereas the concentrations of pentoses remained lower than baseline levels for the duration of the experiment.

### Dipeptides

**Figure 5:**
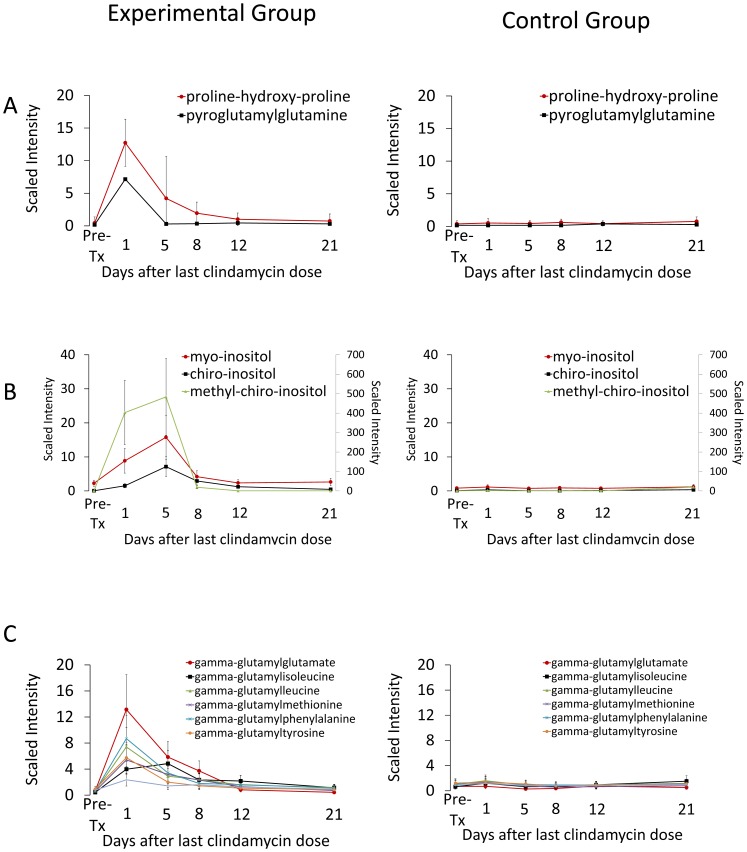
Changes in levels of fecal metabolites of clindamycin-treated mice compared to saline controls for selected compounds that increased or decreased in concentration during clindamycin treatment followed by normalization or substantial return toward baseline within 8 days. Compounds from pathways related to metabolism of (A) dipeptides, (B) inositol isomers and metabolites and (C) gamma-glutamyl amino acids. Results from experimental mice are shown on the left and from control animals on the right. Methyl-chiro-inositol uses the right axis. Metabolites measured in the experimental group are the significantly different (*p*≤0.05) from the pre-treatment levels through day 1 (pyroglutamylglutamine, gamma-glutamylleucine, gamma-glutamylvaline, gamma-glutamylphenylalanine, gamma-glutamyltyrosine), day 5 (proline-hydroxy-proline, myo-inositol, gamma-glutamylglutamate), and day 8 (chiro-inositol, methyl-chiro-inositol, gamma-glutamylmethionine) except gamma-glutamylisoleucine (*p*≤0.10 through day 8). Error bars represent standard error.

Of 124 dipeptides analyzed, 79 (64%) had significantly increased concentrations in clindamycin-treated mice versus controls at one or more time points. However, many of the dipeptides exhibited either persistent or transient increases that did not coincide with the timing of disruption of colonization resistance. Two dipeptides (proline-hydroxy-proline and pyroglutamylglutamine) were identified as potential biomarkers of colonization resistance to be evaluated in future studies based on the magnitude (9–12 fold increase over pre-clindamycin treatment concentrations) and timing of the increase associated with clindamycin treatment (**Figure 5A**). Proline-hydroxy-proline is a dipeptide present in collagen and other connective tissue proteins.

### Inositol Metabolites

Inositol is a cyclohexane with several isomers, including *myo*-inositol and *chiro*-inositol, present in a variety of foods including fruits, grains, and nuts. *Myo*-inositol is a carbohydrate component of the structural lipids phosphatidylinositol phosphates (PIPs) and, in mammals, may be synthesized from glucose. *Myo*-inositol, *chiro*-inositol and especially methyl-*chiro*-inositol (D-pinitol) levels increased markedly during clindamycin treatment, peaking at 5 days after the final clindamycin dose with a rapid decline that coincided with recovery of *in vivo* colonization resistance (**Figure 5B**).

### Gamma-Glutamyl Amino Acids

The gamma-glutamyl amino acids are produced when gamma-glutamyl transpeptidase (GGT) catalyzes the transfer of the gamma-glutamyl moiety of glutathione to amino acids. GGT is present in several mammalian tissues, most notably the liver, and in several bacterial species, as determined using inhibition assays or searches for gene homologes [29], [35]. Several gamma-glutamyl amino acids increased 5 to 19 fold during clindamycin treatment and rapidly declined approaching baseline by day 5 to 8 after the final dose of clindamycin (**Figure 5C**).

### Tryptophan Metabolism

**Figure 6:**
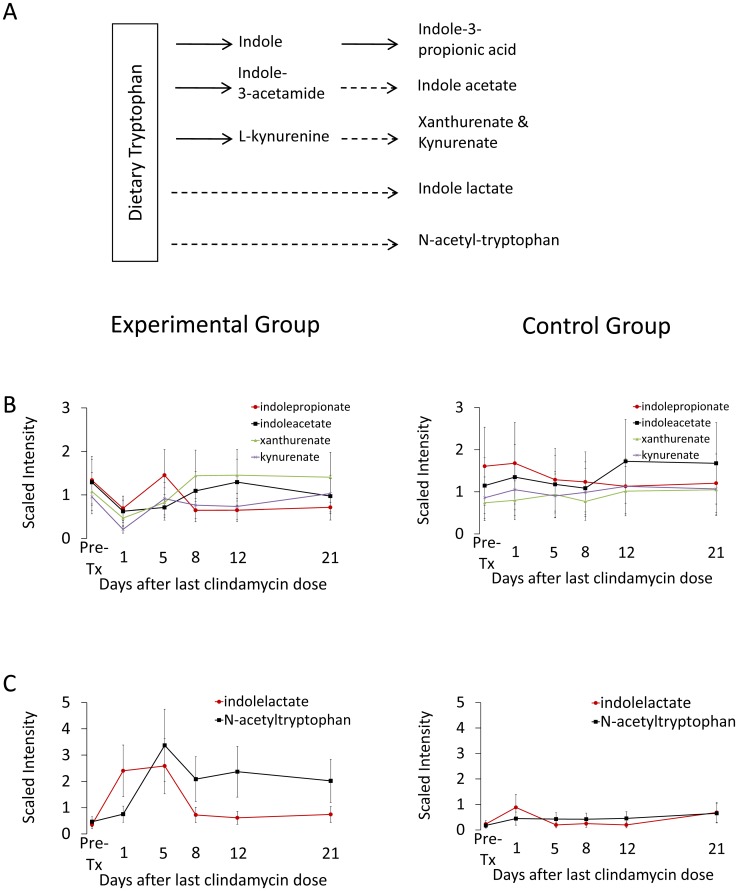
Changes in fecal levels of compounds related to tryptophan metabolism in clindamycin-treated mice compared to control animals. (A) A schematic representation of mammalian metabolism of dietary tryptophan. Biochemical pathways specific to the host are indicated in dashed lines and to the microbiota in solid lines. Fecal levels of compounds with metabolic pathways dependent, at least in part, on the gut microbiota (B) or solely on the host (C). Results from experimental mice are shown on the left and from control animals on the right. Metabolites measured in the experimental group are the significantly different (*p*≤0.05) from the pre-treatment levels through day 1 (indolepropionate, kyurenate), at day 5 (indolelactate) or days 5 – 21 (N-acetyletryptophan). Error bars represent standard error.

The intestinal microbiota synthesize several compounds from tryptophan, an essential amino acid that mammals must absorb from their diet [36]. Tryptophanase produced by enteric bacteria metabolizes tryptophan to indole, with subsequent conversion by other bacterial enzymes to indole-3-proprionic acid (IPA) (**Figure 6A**) [36]. Enteric bacteria also metabolize tryptophan to L-kynurenine which host enzymes convert to xanthurenate and kynurenate. Intestinal anaerobes also convert indole to indole acetate [37]. In germ-free mice, a number of tryptophan metabolites have been shown to be significantly reduced in serum in comparison to conventional control mice [36]. As shown in **Figure 6B**, indole-3-propionate, indole acetate, xanthurenate, and kynurenate, all indole metabolites produced by bacteria, decreased during clindamycin treatment and normalized 5–8 days after the final dose of clindamycin. Synthesis of indole lactate and N-acetyltryptophan from tryptophan relies upon host enzymes for which there are no known bacterial counterparts among the gut microbiome. Levels of these compounds rose 2–3 fold in experimental versus control mice on day 5 following completion of clindamycin exposure (**Figure 6C**), suggesting that in the absence of a robust gut microbiome, tryptophan metabolism may be shunted towards pathways that exist only in the host cellular machinery.

### Correlation of Changes in Intestinal Microbiota and Fecal Metabolites with Restoration of *in vivo* Colonization Resistance after Piperacillin/Tazobactam Treatment

**Figure 7:**
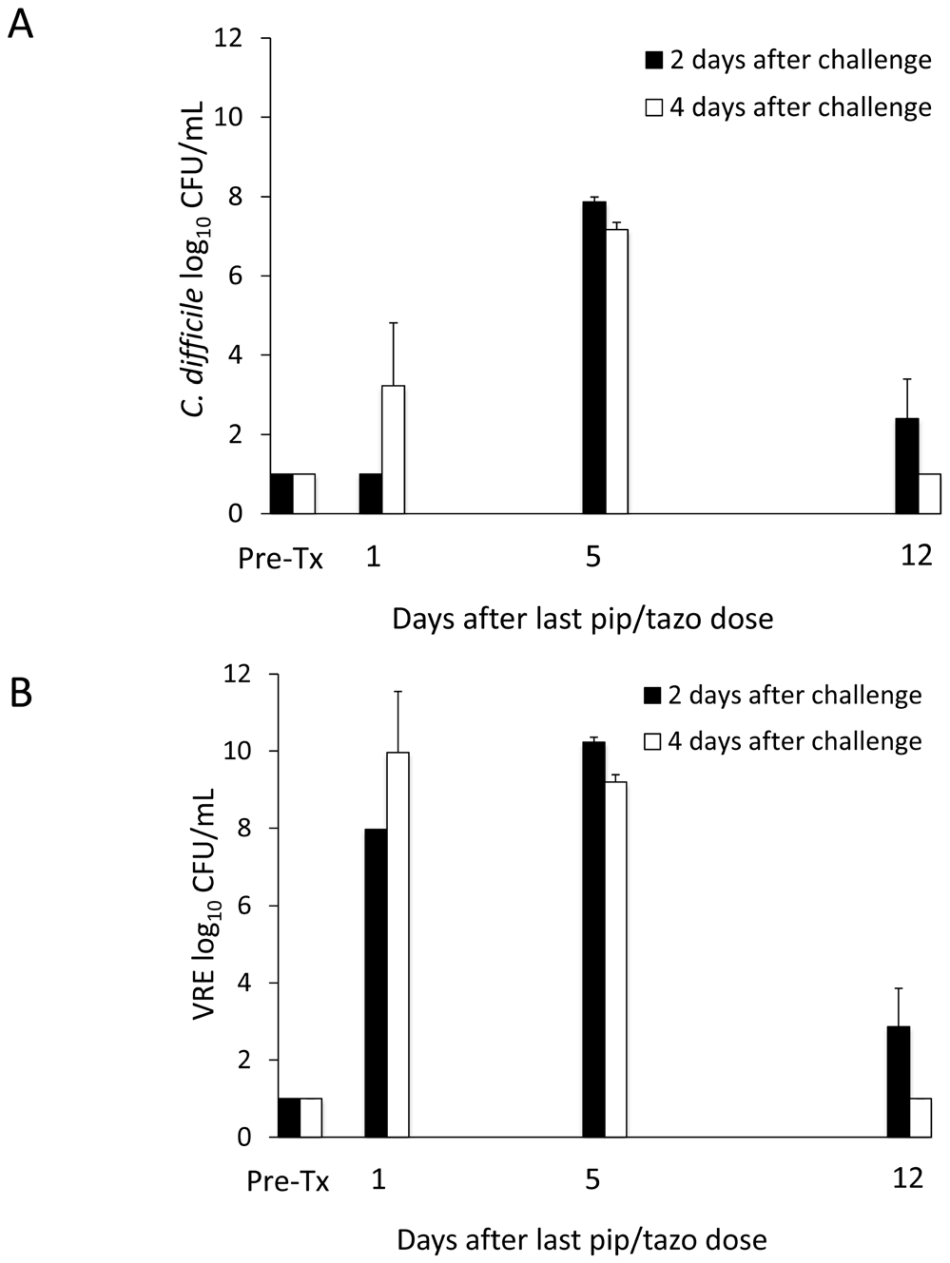
Recovery of *in vivo* colonization resistance over time in piperacillin/tazobactam treated animals. Mice (3–6 per group at each time point) were challenged with 10^4^ colony-forming units of toxigenic *Clostridium difficile* spores (A) or vancomycin-resistant *Enterococci* (VRE) (B) by orogastric gavage either before treatment or 1, 5, or 12 days following treatment with 3 days of daily subcutaneous piperacillin/tazobactam. Concentrations of the pathogens in feces were measured by quantitative cultures 2 days (black bars) and 4 days (white bars) following pathogen challenge. Error bars represent standard error.

**Figure 7** shows the results of the assessment of *in vivo* colonization resistance following exposure to piperacillin/tazobactam. The mean concentration of piperacillin/tazobactam in fecal specimens on day 3 of treatment was 31.4 µg/g (range, 2.0 to 90.2). Mice challenged with *C. difficile* 1 day following completion of piperacillin/tazobactam did not develop colonization, consistent with the fact that the *C. difficile* test strain is susceptible to this agent and low concentrations of piperacillin/tazobactam persist in cecal contents for up to 3 days after treatment [8]. Challenge with *C. difficile* 5 days following treatment resulted in high-density colonization while challenge 12 days later was consistent with restored colonization resistance. Mice challenged with VRE 1 or 5 days after discontinuation of treatment developed high-density colonization, whereas colonization resistance was restored by 12 days after treatment.

**Figure 8:**
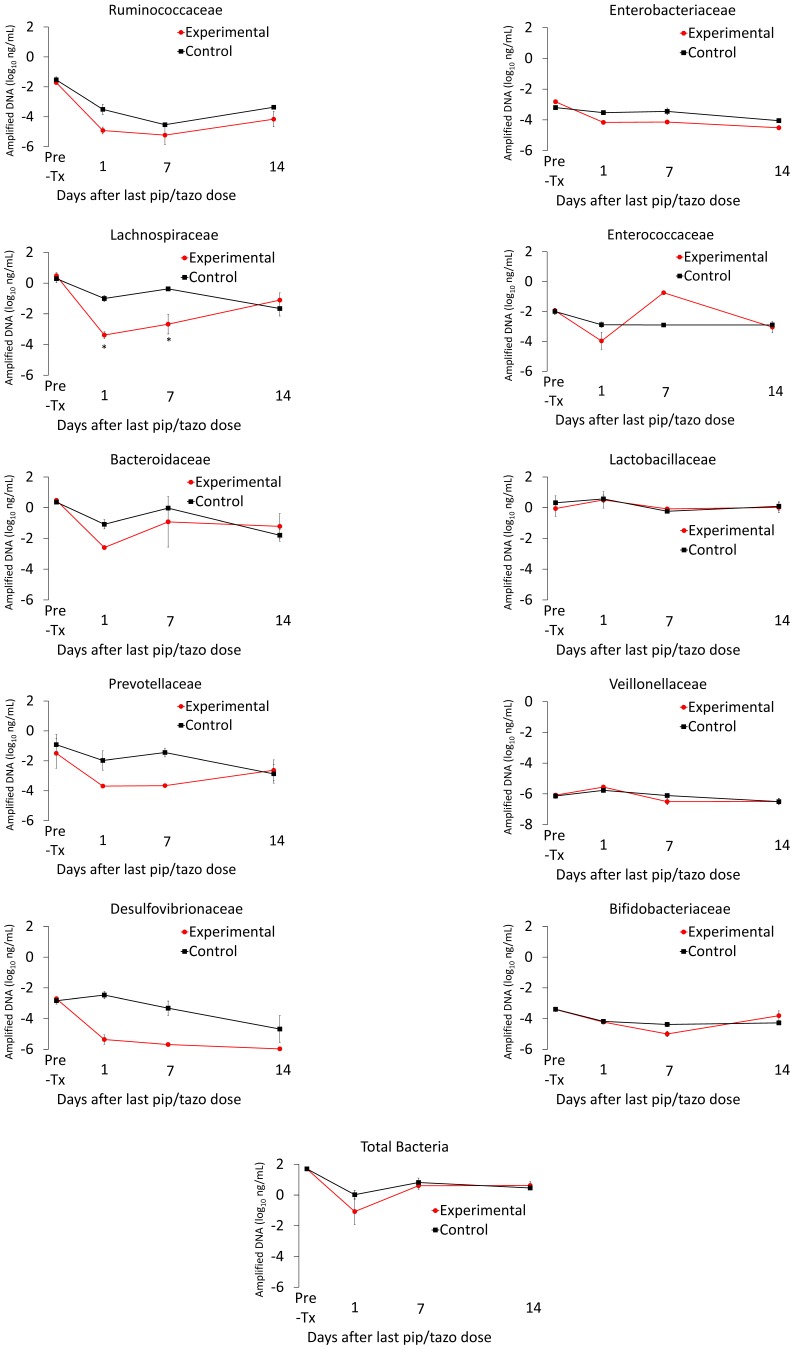
Recovery of the fecal microbiota over time in piperacillin/tazobactam treated animals. Mice received subcutaneous piperacillin/tazobactam (n = 4) or normal saline (n = 4) for 3 days. Quantitative real-time PCR was used to measure fecal bacterial DNA in fecal specimens collected either before treatment or 1, 7 or 14 days following treatment. The y-axis shows the quantity of amplified DNA detected per 1 ng of template DNA. Red circles, mean values for clindamycin-treated mice. Black squares, mean values for control mice. * *p*<0.05. Symbols indicate differences between the experimental and control groups for individual time points. Error bars represent standard error.

**Figure 8** shows the results of qPCR analysis of changes in the microbiota during and after piperacillin/tazobactam treatment. Total bacterial DNA levels declined during treatment with piperacillin/tazobactam, consistent with its broad effect on the gut microbiota (i.e., suppression of indigenous enterococci and facultative gram-negative bacilli in addition to anaerobes). Compared to control mice, piperacillin/tazobactam suppressed fecal bacterial DNA from the family *Lachnospiraceae* with a return to baseline concentrations coinciding with recovery of *in vivo* colonization resistance. Similar to clindamycin-treated animals, bacteria from the Families *Lactobacillaceae, Veillonellaceae* and *Bifidobacteriaceae* were largely unaffected by piperacillin/tazobactam treatment.

**Figure 9:**
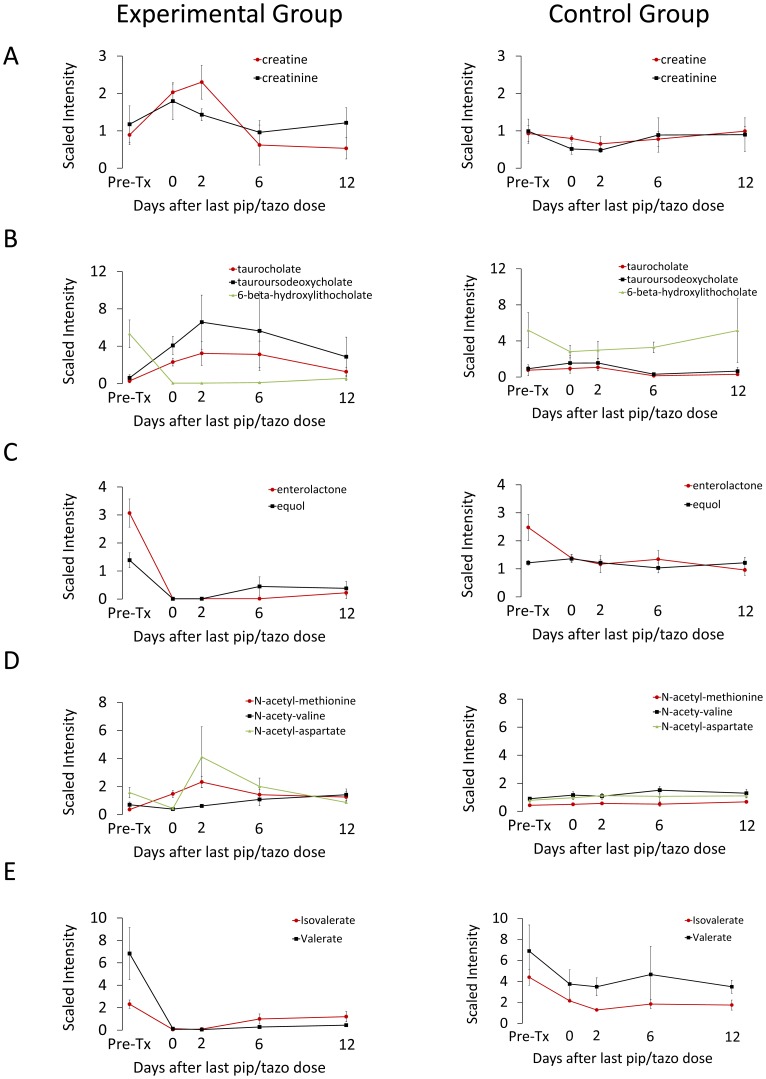
Changes in levels of fecal metabolites of piperacillin/tazobactam-treated mice compared to saline controls for selected compounds that exhibited a sustained increase or decrease after clindamycin treatment. Compounds from pathways related to metabolism of (A) creatinine, (B) bile salts; (C) phytoestrogens (D) N-acetylated amino acids and (E) short-chain fatty acids. Results from experimental mice are shown on the left and from control animals on the right. Metabolites measured in the experimental group are the significantly different (*p*≤0.05) from the pre-treatment levels through day 0 (N-acety-valine, N-acetyl-aspartate), day 2 (creatine, creatinine, equol, N-acetyl-methionine, N-acetyl-aspartate), at day 2 (taurocholate,) and days 6 and 12 (6-beta-hydroxylithocholate, valerate). Error bars represent standard error.

**Figure 10:**
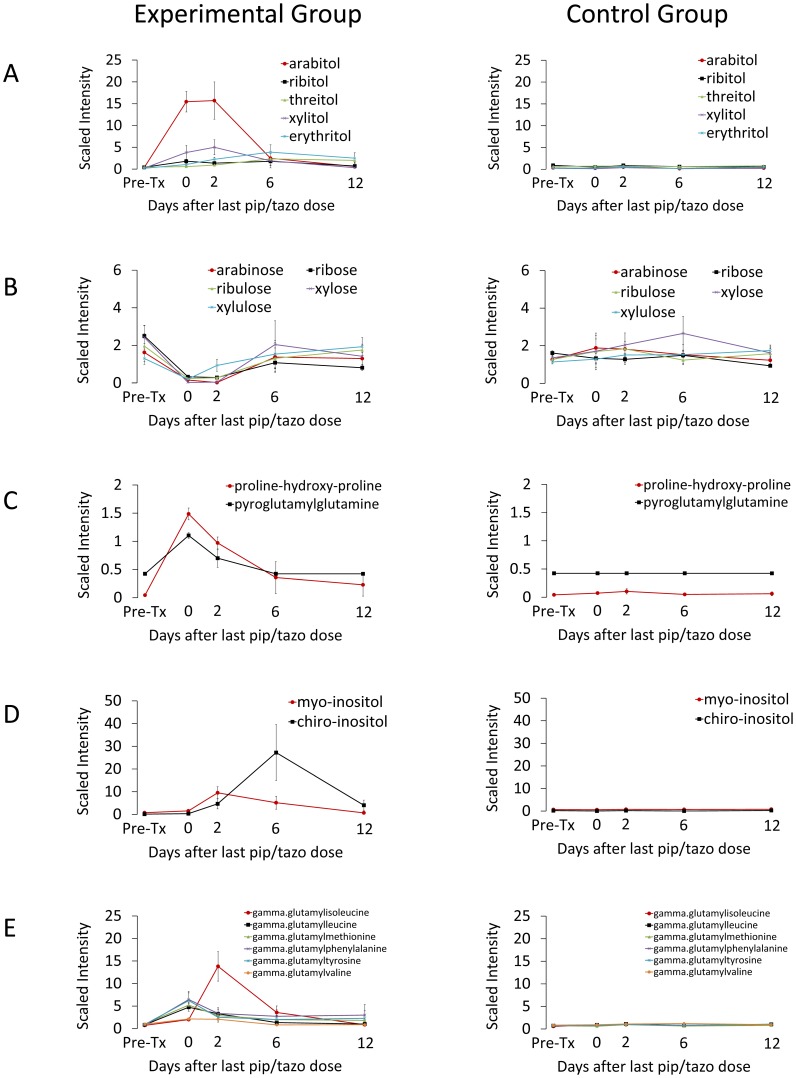
Changes in levels of fecal metabolites of piperacillin/tazobactam-treated mice compared to saline controls for selected compounds that increased or decreased in concentration during treatment followed by normalization or substantial return to baseline within 8 days. Compounds from pathways related to metabolism of (A) 4- and 5-carbon alcohols and (B) sugars, (C) dipeptides, (D) inositol isomers and metabolites and (E) gamma-glutamyl amino acids. Results from experimental mice are shown on the left and from control animals on the right. Metabolites measured in the experimental group are the significantly different (*p*≤0.05) from the pre-treatment levels through day 0 (ribitol, xylulose, pyroglutamylglutamine, gamma-glutamylvaline, day 6 (arabitol, erythritol, chiro-inositol, gamma-glutamylisoleucine, gamma-glutamylmethionine), at day 6 (threitol) or through day 2 (all other metabolites). Error bars represent standard error.

Piperacillin/tazobactam treatment resulted in patterns of alteration in fecal metabolites similar to the changes associated with clindamycin. **Figure 9** shows data for several metabolic compounds that were not considered potential biomarkers of colonization resistance based upon a sustained increase or decrease in experimental versus control mice. **Figure 10** shows data for several metabolic compounds that increased or decreased in concentration during piperacillin/tazobactam treatment followed by normalization or substantial return toward baseline within 6 days after the final antibiotic dose. These compounds were considered potential biomarkers of colonization resistance based upon the correlation between their recovery and the recovery of *in vivo* colonization resistance to both piperacillin/tazobactam and clindamycin.

## Discussion

Although essential for treatment of infections, antibiotics often result in unintended adverse consequences due to disruption of the indigenous microbiota of the host. Consistent with previous studies [7], [8], [10], we demonstrated that clindamycin and piperacillin/tazobactam treatment causes profound alteration of the intestinal microbiota, with some anaerobic bacterial families failing to recover to baseline levels by 21 days after treatment. However, the rapid restoration of colonization resistance against *C. difficile* and VRE by 5 to 12 days after treatment also highlights the resilience of the microbiota. By correlating the timing of functional recovery of colonization resistance with changes in concentrations of fecal metabolites, we identified a number of potential biomarkers that could provide useful indicators of intact or disrupted colonization resistance during and after antibiotic treatment. Such biomarkers could be used to determine the susceptibility of patients to intestinal colonization by healthcare-associated pathogens including *C. difficile*. This information could be useful to guide infection prevention efforts and to evaluate the impact of antimicrobials and probiotics on colonization resistance.

Our findings are consistent with recent evidence that bacteria from the phylum *Firmucutes* may play an important role in colonization resistance. As noted previously, administration of isolates from the bacterial family *Lachnospiraceae* (clostridial cluster XIVa) and a mixture of bacteria including Anaerostipes sp. nov. (clostridial cluster XIVa) have been shown to be effective in partially or completely restoring colonization resistance to *C. difficile* in mice [9], [11]. In hospitalized patients, a reduction in the abundance of the family *Clostridiales* (clostridial cluster XI) was independently associated with increased risk of nosocomial *C. difficile* infection [38]. Another study found that patients with *C. difficile* infection or nosocomial diarrhea had significant depletion of bacteria from the *Lachnospiraceae* and *Ruminococcaceae* families and butyrate-producing anaerobic fermenters [39]. Here, we demonstrated that recovery bacteria from the families *Lachnospiraceae* and *Ruminococcaceae* (phylum *Firmicutes,* order *Clostridiales*) corresponded directly with the timing of recovery of *in vivo* colonization resistance. In contrast, bacteria from the families *Bacteroidaceae*, *Prevotellaceae* and *Desulfovirionaceae* were suppressed by clindamycin but did not recover to baseline levels within 21 days after the final clindamycin dose. As has been described previously, concentrations of bacteria from the families *Enterococcaceae* and *Enterobacteriaceae* increased during clindamycin treatment and decreased to near baseline levels by 8–12 days after the final day of treatment, providing potential indicators of colonization resistance recovery that can be easily measured using standard culture methods [6]–[8], [10], [11], [39].

Clindamycin treatment caused marked changes in metabolites present in fecal specimens. Of 484 compounds measured, 35% changed significantly in concentration during clindamycin treatment, confirming the involvement of anaerobic intestinal bacteria in their metabolism. However, many of these compounds remained significantly higher or lower than their baseline levels well beyond the recovery of *in vivo* colonization resistance. For example, concentrations of the phyotoestrogen metabolites enterolactone and equol, the secondary bile salt 6-beta-hydroxylithocholate, the minor SCFAs valerate and isovalerate as well as an N-acetylated amino acid, aspartate, were suppressed during clindamycin treatment and remained low. In contrast, the concentrations of 3 other N-acetylated amino acids, isoleucine, methionine and valine, increased and remained high for 21 days after clindamycin treatment. A likely explanation for the prolonged alteration of these compounds is that their metabolism is dependent on bacterial species whose levels are altered for a prolonged period after clindamycin treatment. None of these compounds were identified as potential biomarkers of mediators of colonization resistance because changes in their relative concentrations did not change with recovery of *in vivo* colonization resistance.

We also identified several compounds with marked increase or decrease in concentration during clindamycin treatment followed by normalization or substantial return toward baseline within 8 days. The recovery of these compounds and those in closely related metabolic pathways correlated with recovery of colonization resistance, identifying them as potential biomarkers and/or mediators of colonization resistance. Several of these potential biomarkers were intermediates in carbohydrate or protein metabolism that increased during clindamycin treatment (pentitols, gamma-glutamyl amino acids, urea, cadaverine, saccharapine, and inositol metabolites or isomers), presumably due to loss of metabolic digestion by anaerobic microbiota. The increase in the dipeptides (proline-hydroxy-proline and pyroglutamylglutamine) during clindamycin treatment is notable because previous studies identified the dipeptide beta-aspartylglycine as a potential indicator of colonization resistance (i.e., present in feces of germ-free or antibiotic-treated mice but not in the presence of intact indigenous microbiota) [40].

Tryptophan metabolites containing indole are a final potential group of compounds identified as potential biomarkers of colonization resistance. Indole-3-propionate and kynurenate decreased significantly during clindamycin treatment with normalization by 5–8 days after treatment. These data are consistent with recent studies demonstrating that indole-containing metabolites derived from tryptophan are significantly reduced in blood of germ-free mice [36].

The similarity of findings for piperacillin/tazobactam and clindamycin suggest that the potential biomarkers of colonization resistance identified will be applicable to multiple classes of antibiotics. Piperacillin/tazobactam and clindamycin both suppresses anaerobic intestinal microbiota and disrupt colonization resistance [7]–[8]. Piperacillin/tazobactam also suppresses indigenous enterococci and facultative gram-negative bacilli, but these organisms make up a minor proportion of the total microbiota of healthy adults and have not been identified as important for colonization resistance [7]–[8].

Our finding that antibiotic treatment dramatically alters fecal metabolites is consistent with other recent studies that examined the effect of antibiotics on fecal or urinary metabolites using high resolution ^1^H nuclear magnetic resonance (NMR) spectroscopic based profiling [41]–[44], [11], [45]. Yap *et al.* demonstrated that oral vancomycin treatment resulted in reduced fecal excretion of amino acids, SCFAs and uracil as well as increased levels of choline and oligosaccharides [41]. Romick-Rosendale *et al.* demonstrated that 8 metabolites changed significantly in fecal extracts of mice treated with enrofloxacin, including reductions in SCFAs, decreased amino acids, and increased urea [42]. Swann *et al.* demonstrated that penicillin and streptomycin-induced alteration of the intestinal microbiota of rats was associated with reduced fecal levels of SCFAs, alanine, lactate, methionine, and succinate and increased fecal levels of taurine, tryptophan, asparagine, choline, and oligosaccharides [43]. Using a methodology comparable to ours, Theriot *et al.* reported very similar metabolite changes associated with the broad-spectrum antibiotic cefoperazone, including elevation of sugar alcohols and primary bile acids, and decreases in secondary bile acids and short chain fatty acids [44]. Moreover, it was demonstrated that *C. difficile* was able to exploit these antibiotic-induced metabolic changes to colonize the intestinal tract, including use of the primary bile acid taurocholate for germination and sugar alcohols and other carbon sources for growth [44]. Similarly, Ng *et al*. demonstrated that antibiotic treatment increased levels of free sialic acid in mice, providing a potential source of nutritional support for *C. difficile* [45]. Finally, Lawley *et al.* have also demonstrated that clindamycin-induced disruption of colonization resistance was associated with reduction in SCFAs [11].

Our study has some limitations. First, the *in vivo* colonization resistance assessment included only one strain each of VRE and *C. difficile*. However, our findings are consistent with previous studies in which it was demonstrated that recovery of colonization resistance occurred within days after discontinuation of clindamycin or other antibiotics, including for toxigenic *C. difficile* strains [7], [8], [10]. Second, in our mouse model of colonization resistance, mice become colonized with high concentrations of toxigenic *C. difficile* without developing signs of illness or mortality. Thus, we cannot exclude the possibility that the metabolic changes do not reflect changes associated with more prolonged or profound disruption of the microbiota which has been associated with development of *C. difficile* disease in mice [11], [44], [46], [47]. However, the metabolic changes associated with colonization in our model were very similar to the changes associated with susceptibility to disease in the model of Theriot *et al*. [44]. In addition, the levels of bacteria detected per gram of stool in mice is typical of levels measured in humans infected with *C. difficile* or colonized with VRE [48], [49]. Third, because our analysis of the microbiota included only a limited number of bacterial groups, further studies are needed that include more extensive analysis of the microbiota. Additional studies are also needed to determine which bacterial species are responsible for production of specific metabolites. Given the degree of functional redundancy of the intestinal microbiota, it is likely that multiple families of bacteria may be able to carry out the metabolic conversions required to produce the metabolites identified here. Fourth, mice and humans differ in metabolism and elimination of antibiotics. However, the measured concentrations of both antibiotics in feces of mice were consistent with levels measured in previous studies in humans. Fifth, antibiotics that alter the anaerobic microbiota result in a change in the consistency of feces (i.e., softer and larger pellets) in the mice used in these experiments without causing overt diarrhea. Thus, we cannot rule out the possibility that a wash out or dilution effect caused some reduction in the concentrations of microbiota and fecal metabolites. However, the fact that some bacterial groups and metabolites increased while others decreased during antibiotic treatment is not consistent with a significant wash out effect. Sixth, although many compounds were analyzed in the current study, it is possible that the methods used might miss some important potential biomarkers or mediators of colonization resistance. For example, it has been proposed that the predominant SCFAs (acetate, butyrate, and propionate) may contribute to colonization resistance [11], [12], but they were not measured here because they were below the molecular weight cutoff of the methods used or were lost during extraction. Seventh, the conclusions that can be drawn from the piperacillin/tazobactam experiments are limited by the small numbers of mice included in each group. However, the findings for time to recovery of colonization resistance are consistent with a previous study [7]. Finally, the non-targeted metabolic profiling analyses conducted in the current study provide a relative measure of changes in fecal metabolites. Although the consistency of the changes in fecal metabolites associated with 2 antibiotic classes provides support for their potential utility as biomarkers, additional studies that include quantitative measurements of the potential biomarkers will be needed.

## Supporting Information

Table S1 **Amplicons specific to each primer set.** Bacterial targets for each primer set were determined *in silico* using TestProbe from SILVA-ARB (www.silva-arb.de) [23]. The results for each primer set were concatenated to identify amplicons from known bacterial species. Sequences representing 1% or more of the amplicons are shown.
(XLSX)

Table S2 **Changes in mouse fecal metabolites following clindamycin exposure.** Column C lists the metabolites analyzed, along with their pathway and links to databases (columns A-B & F-G, respectively). The effect of clindamycin treatment is expressed as a ratio the metabolites detected in the fecal material of treated animals *vs.* control animals at each time point (columns K-O). Column H describes patterns observed include no change in treated mice compared to controls (1), a sustained change compared to controls (2), an initial changes with a substantial return to baseline within 8 days (3) or a variable response (4). Metabolites with a significant difference at baseline (prior to initiation of treatment) are coded a as 0. For patterns 2 & 3, an increase in the proportion of metabolites from the treated *vs.* control group is coded as 0 and a decrease as 1 (Column I). Results from statistical analysis are included in the second worksheet.
(XLSX)
